# Identification of Differentially Expressed Kinase and Screening Potential Anticancer Drugs in Papillary Thyroid Carcinoma

**DOI:** 10.1155/2016/2832980

**Published:** 2016-09-15

**Authors:** Huairong Zhang, Bo Gao, Bingyin Shi

**Affiliations:** ^1^Department of Endocrinology, First Affiliated Hospital of Xi'an Jiaotong University, Xi'an, Shaanxi 710061, China; ^2^Department of General Surgery, First Affiliated Hospital of Harbin Medical University, Harbin, Heilongjiang 15001, China

## Abstract

*Aim*. We aim to identify protein kinases involved in the pathophysiology of papillary thyroid carcinoma (PTC) in order to provide potential therapeutic targets for kinase inhibitors and unfold possible molecular mechanisms.* Materials and Methods*. The gene expression profile of GSE27155 was analyzed to identify differentially expressed genes and mapped onto human protein kinases database. Correlation of kinases with PTC was addressed by systematic literature search, GO and KEGG pathway analysis.* Results*. The functional enrichment analysis indicated that “mitogen-activated protein kinases pathway” expression was extremely enriched, followed by “neurotrophin signaling pathway,” “focal adhesion,” and “GnRH signaling pathway.” MAPK, SRC, PDGFRa, ErbB, and EGFR were significantly regulated to correct these pathways. Kinases investigated by the literature on carcinoma were considered to be potential novel molecular therapeutic target in PTC and application of corresponding kinase inhibitors could be possible therapeutic tool.* Conclusion*. SRC, MAPK, and EGFR were the most important differentially expressed kinases in PTC. Combined inhibitors may have high efficacy in PTC treatment by targeting these kinases.

## 1. Introduction

Thyroid cancer, particularly papillary thyroid carcinoma, is considered to be one of the most common malignancies. Its incidence increases in differed geographic regions of the world in the past decade. Increase in incidence of thyroid cancer is one of the top malignancies in the United States [1]. Rise in incidence observed may partly be accounted for improvement in diagnosis. It is indicated that papillary thyroid carcinoma is attributed to the entire increase significantly by separated researches [2, 3]. Although the thyroidectomy, thyroid-stimulating hormone suppression therapy, and radioiodine remnant ablation (RAI) ameliorate the disease-free survival, a group of patients still cannot benefit from the traditional therapy. 5% of patients with distant metastasis are refractory to RAI [4]. It is believed that the tumor cells lost their ability in uptake of the iodide and side effects of ^131^I therapy of salivary gland dysfunction (>40%), abnormally dry eyes (25%), and transient fertility reduction (20%) affect the patient living quality seriously [5]. Besides, patients are contraindicated to thyroidectomy who suffer from cardiac or respiratory disease, dialysis-dependent renal failure, anticoagulant therapy, obstructive sleep apnea, mental impairment, thyrotoxicosis, and morbid obesity [6]. The postoperative complications of surgery are quite common including hypocalcemia (20%–30%) and recurrent laryngeal nerve injury (5%–11%) and risk for nerve injury increased significantly by reoperation [7]. Due to the limits of traditional therapy, more efforts are recalled for new drugs development with higher efficiency and fewer side effects. It is protein kinases that function as core in signaling pathways participating in tumor proliferation, invasion, metastasis, and tumor microenvironment formation in major types of tumors, including thyroid cancer pathogenesis.

It has been identified that in papillary thyroid carcinoma several molecular changes exist: rearranged during transfection (RET)/papillary thyroid carcinoma gene rearrangements, BRAF (B-RAF protooncogene, serine/threonine kinase) gene mutations, RAS (rat sarcoma) mutations, and vascular endothelial growth factor receptor 2 angiogenesis pathways activation. BRAF oncogene mutation occurred in approximately 45% to 70% of patients with papillary thyroid carcinoma and VEGF overexpression is frequently found in tumors that originated in the thyroid [8–11]. In the landmark DECISION study, Sorafenib, a multikinase inhibitor of RET/RAS/RAF pathway, VEGF receptors 2 and 3, improved the progression-free survival by 3 months, from 5.8 months to 10.8 months, compared with placebo [12]. It emerged as a potentially effective option and approved by Food and Drug Administration (FDA) as a receptor tyrosine kinase inhibitor as treatment of differentiated thyroid cancer refractory to RAI.

We aimed to investigate the protein kinase expression difference between papillary thyroid carcinoma and normal thyroid and possible molecular mechanisms underlying PTC. We try to provide valuable information on PTC associated protein kinases for potential therapeutic targets.

## 2. Method

### 2.1. Identify Differentially Expressed Protein Kinases in PTC

We searched in the NCBI (National Center for Biotechnology Information) Gene Expression Omnibus (GEO) database (https://www.ncbi.nlm.nih.gov/geo/) to retrieve the genome-wide expression profile of human papillary thyroid carcinoma. The original dataset from GSE27155 including 51 human specimens of PTC and 4 specimens of normal thyroid tissues was downloaded and array from the Affymetrix platform was processed. To discover the differential gene expression the significance analysis of microarrays (SAM) was applied. False discovery rate (FDR) less than 0.05 was considered to present significant difference. All the differentially expressed genes were mapped onto The Human Protein Kinase Reference Database to establish differentially expressed kinases in PTC. Then we conducted systematic screening of the literature in PubMed for researches concerning the association between papillary thyroid carcinoma and protein kinases.

### 2.2. Database-Based GO and Pathway Enrichment Analysis and Literature Review

Each protein kinase's corresponding GO term or ID in the GO database was validated by AmiGO search engine. Gene ontology (GO) enrichment analysis was used to evaluate the potential function for the differentially expressed genes. The pathway was analyzed by the Kyoto Encyclopedia of Genes and Genomes (KEGG) database. To obtain putatively valuable new kinase targets for the treatment of PTC, we conducted a systematic literature screening of all the protein kinases. The keywords in the title and abstracts “each protein kinase,” “cancer,” and “papillary thyroid carcinoma” were used. Finally, we drew the picture of interactions among kinases, signaling pathways, and GO terms.

### 2.3. Screening Kinase Inhibitors for Predicted Kinases Involved in PTC

To obtain new potentially valuable kinase inhibitors for PTC therapy, we systematically screened all available multikinase and specific kinase inhibitors (http://www.selleckchem.com/). We than retrieved the number of publications related to each kinase inhibitor and carcinoma.

## 3. Results

### 3.1. Establishment of Differentially Expressed Protein Kinases

There are 518 human protein kinases in total in the protein kinase database. Amongst them, 110 kinases were predicted to be related to papillary thyroid carcinoma in our network. We tried to delineate a network of protein kinases engaged in PTC. Several kinase families exhibited correlation to pathophysiological process of PTC via the GO database. Here, we presented a map of these kinases to visualize their relationship in biological process.

### 3.2. Functional Analysis and Literature Review

**Figure 1:**
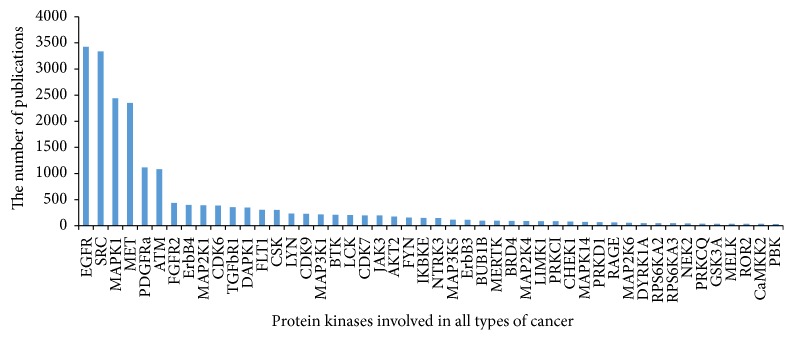


**Figure 2:**
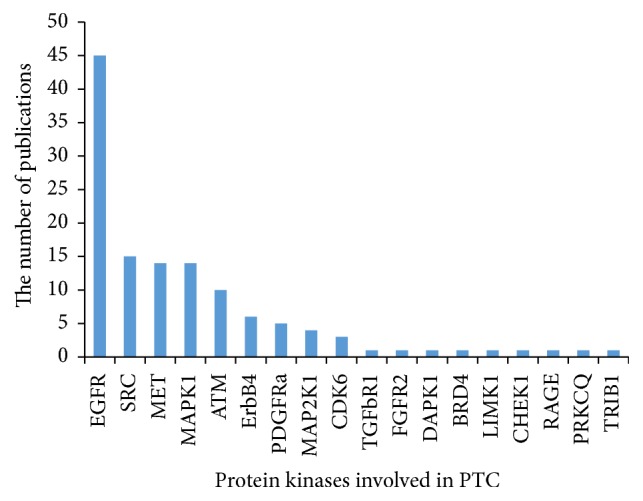


**Figure 3:**
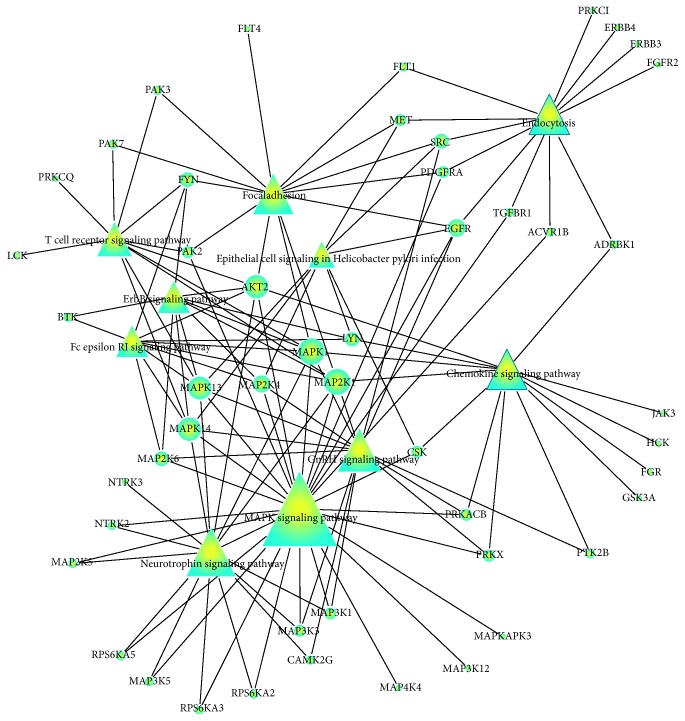
Kinases to pathway interactions network in PTC.

**Figure 4:**
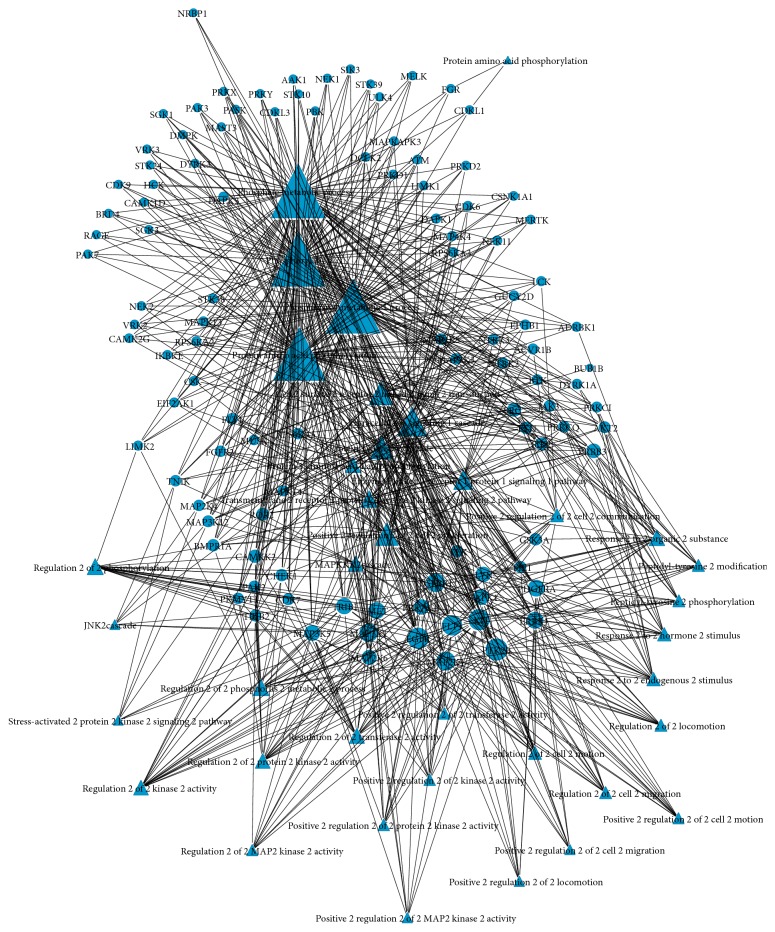
Kinases and GO network in PTC.

GO and KEGG enrichment analyses were conducted in the differentially expressed kinases. Kinases with a result of less than 0.05 in GO terms and pathways with FDR-value were considered to be the most valuable possible targets and were applied for further analysis. Study in different kinds of cancer mainly focused on the roles of SRC (v-src sarcoma (Schmidt-Ruppin A-2) viral oncogene homolog (avian)), MAPK (mitogen-activated protein kinase), MET (met protooncogene (hepatocyte growth factor receptor)), ATM (ataxia telangiectasia mutated), PDGFRa (platelet-derived growth factor receptor, alpha polypeptide), ErbB (v-erb-a erythroblastic leukemia viral oncogene homolog), MAP2 K1 (bromodomain containing 4), TGFbR1 (transforming growth factor, beta receptor 1), and CDK6 (cyclin-dependent kinase 6) (shown in Figure 1). Coincidently, the above-predicted kinases were confirmed with differentially protein kinases expression investigated in PTC patients (Figure 2). 39 GO terms were annotated and presented as common biological process among all these kinases (Table 1). Finally, we built networks to investigate the relationship among kinases, signaling pathways, and GO terms (Figures 3 and 4).

### 3.3. Potential Novel Kinase Inhibitors for PTC

To investigate novel kinase inhibitors for the treatment of PTC, we conducted a literature review of the cancer-related research including 20 focused kinases involved in our network. The publication number could indicate the extent of the research on kinase inhibitor use in cancer treatment (Table 2).

## 4. Discussion

Tyrosine kinases, classified as receptor tyrosine kinase (RTK) and nonreceptor tyrosine kinase (nRTK), are essential mediators of signaling pathways through catalyzing phosphorylation of selecting tyrosine residues with ATP, leading to cell proliferation differentiation, migration, and metabolism. Recently, it has come in vogue to use tyrosine kinases inhibitors as conceivable anticancer drug and several tyrosine kinase inhibitors (TKIs) have been approved by FDA in neoplastic human disease, particularly in the thyroid cancer. The largest subfamily of nRTKs, Src family kinase (SFK), was closely related to PTC indicated by our network. It has been found that Src and Lyn are expressed in thyroid cancer cells and Src is overexpressed and activated in thyroid cancer [13]. Src also plays an important role in tumor metastasis due to its function in regulation of cytoskeleton, cell migration, adhesion, and invasion through phosphorylation of focal adhesion kinase (FAK), a component of focal adhesion complexes [14–16]. BTK and HCK expressions are upregulated in invasive thyroid cancer compared to matched normal group, which is similar to Src. Dasatinib, a FDA-approved SFK inhibitor, can block PTC tumor growth by more than 90% and significantly inhibited metastasis [13]. Apart from Src family, several kinase families were also annotated in the GO analysis: Bruton agammaglobulinemia tyrosine kinase (BTK), Janus Kinase 3 (JAK3), and c-src tyrosine kinase (CSK). However, the role of theses kinase families in PTC has not been reported yet and requires further elucidation.

It is epidermal growth factor family member, one of the receptor tyrosine kinase (RTK) families, that not only induces cell growth but also contributes to cell migration and proliferation via its downstream signaling pathways such as mitogen-activated protein kinase (MAPK) and phosphoinositide 3-kinase (PI3K)/AKT pathways, which leads to cell migration, adhesion, and proliferation [17]. Several independent studies documented that EGFR mRNA expression is increased in PTC compared with benign thyroid lesions and EGFR protein expression is upregulated simultaneously [18–21]. Besides, EGFR expression was noted to possess prognostic value for that its expression was significantly associated with lymph node metastasis in a retrospective analysis of 168 patients with PTC [22]. Platelet-derived growth factor (PDGF)/PDGF receptor (PDGFR) system, another member of RTK class III, contributes to tumor formation, cell survival, growth, and proliferation [23–25]. Previous studies presented that PDGFR-*α* was upregulated at both mRNA and protein levels in thyroid carcinoma cell lines compared with benign tissues of thyroid nodular hyperplasia [25]. It was revealed that association of PDGFR-*α* with aggressive and lymph node metastatic phenotype in PTC was achieved through both the MAPK/ERK and PI3K/Akt pathways [26].

MET gene is one of the useful molecular markers for PTC [27] and elevated at RNA and protein level in frozen thyroid tissue samples and fine-needle aspiration biopsy [28]. However, no expression of c-met and its ligand hepatocyte growth factor/scatter factor (HGF/SF) could be detected in normal thyroid tissue [29]. MET could strengthen malignancy by its interaction with vascular endothelial growth factor receptor (VEGFR) which induces angiogenesis [30]. In the past decade, several MET kinase inhibitors have been developed, for example, the Tivantinib. Tivantinib impedes ligand-mediated MET autophosphorylation to reduce invasion, metastasis, and proliferation [31, 32]. Several Phase II trials on Tivantinib have been in process treating different tumor types, in which Tivantinib treatment showed antitumor effects with monotherapy in microphthalmia transcription factor family associated tumors [33] and prolonged progression-free survival in nonsmall-cell lung cancer [34]. MET may also be exhibited to be overexpressed in PTC with extremely limited investigation done in thyroid carcinoma. Cabozantinib is another FDA-approved tyrosine kinase inhibitor targeting three important pathways: MET, vascular endothelial growth factor (VEGF), and rearranged during transfection (RET) for the treatment of metastatic medullary thyroid cancer. It has shown significant effects in prolongation of progression-free survival with acceptable safety profile [35]. Based on the above trials, inhibiting of MET pathway (or in combination with other kinase inhibitors) is quite a possible way to improve the prognosis of PTC.

Mitogen-activated protein kinase (MAPK) is regarded as a member of serine/threonine protein kinases and a canonical pathway activated by BRAF, RET, or TRK and RAS mutations through Ras-Raf-MEK-ERK cascade in PTCs [36–38]. MAPK members act as key regulators for cell growth, proliferation, and differentiation during cancer progression. Previous studies have reported that matrix metalloproteinases (MMPs) were modulated according to the intensity of MAPK pathway activation which partly explained the mechanism of increased propensity of tumor invasion in PTC patients carrying BRAF mutation [39, 40]. The phosphorylation status of MAPK molecule, p38 MAPK signaling pathway, could be achieved by high expression of family with sequence similarity 172, member A (FAM172A) in human PTC which induced cell proliferation. However, the effects following MAPK molecule phosphorylation can be attenuated markedly by inhibitor of p38 MAPK, SB202190 [41]. Other two types of the mitogen-activated protein kinase (MAPK) cascade, namely, MAPK kinase (MAPKK/MEK) and MAPK kinase kinase (MAPKKK/MEKK) which play important roles in cell growth, were included in our network. It is well known that these two upstream molecules of MAPKs regulate cell proliferation and apoptosis by activating MAPK pathways [42]. Strikingly, inhibitors of MEK (PD0325901) may also contribute to restoration of tumor cells RAI uptake by recovery expression of Na^+^/I^−^ symporter (NIS) protein [43]. The propensity of maintaining stability of NIS by MEK was proved again in human breast cancers [44].

Tumors are characterized for uncontrolled cell division. Cyclin-dependent kinases (CDKs) responsible for controlling cell cycle were expected to become an effective therapeutic target. Various evidences point to a crucial role of an aberrant cyclin D1-CDK4/6 complex in initiation and progression of cancers. Cyclin D1 expressed in about 30% of PTC carcinoma [45] and its overexpression correlate with metastasis of PTC [46]. P27^KIP1^, a CDK inhibitor that could impair the activity of cyclin-CDK complex, was found to be reduced in metastatic forms of PTC [47, 48]. Therefore, CDKs are attractive set of targets for novel anticancer drug development.

To conclude, protein kinases play essential roles in controlling cellular growth, cell proliferation, and cell death and have been found to participate in human neoplastic diseases. Our network presented potential kinases involved in several aspects of papillary thyroid carcinoma development including invasion, metastasis, progression, and sensitivity to RAI. Many of the kinase inhibitors are undergoing clinical trials while several have already been approved for treatment of PTC and/or other types of cancer. In addition to the traditional kinases applied in PTC, we provided more kinases which have not been equivocally investigated but are potentially effective options in the treatment of PTC. Therefore, targeting abnormal activation of tyrosine kinases is a promising way to treat PTC.

## Figures and Tables

**Table 1:** The functions of the most discussed protein kinases involved in PTC.

Function	Kinases
Protein amino acid phosphorylation	EGFR, MAPK1, MEK, MEKK, MEKKK, CDKL1, PDGFRA, FGFR2, ERBB4, ERBB3, SRC, TGFBR1, MET, ATM, CSK, LYN, CDK9, CDK6, CDK7, LCK, HCK, FLT4, FYN, JAK3
Phosphorylation	BTK, MEKKK, MEKK, EGFR, MAPK1, PDGFRA, FGFR2, ERBB4, ERBB3, EPHB1, SRC, TGFBR1, MET, ATM, CSK, CDK6, LCK, HCK
Phosphorus metabolic process	BTK, MEK, MEKK, EGFR, MAPK1 PDGFRA, FGFR2, ERBB4, ERBB3, EPHB1, SRC, TGFBR1, MET, ATM, CSK, LYN, CDK6, LCK, FYN, JAK3
Phosphate metabolic process	BTK, MEKK, MEK, EGFR, MAPK, PDGFRA, FGFR2, FGR, ERBB4, ERBB3, EPHB1, SRC, TGFBR1, MET, ATM, NTRK3, NTRK2, DYRK1A, CSK, LYN, CDK9, CDK6, CDK7, LCK, HCK, FYN JAK3
Protein kinase cascade	ERBB3, SRC, BTK, MEKK, MEK, EGFR, TGFBR1, MET, MAPK, FYN, JAK3
Intracellular signaling cascade	BTK, MEKK, CSK, MEK, EGFR, LYN, CDK, MEKKK, MAPK1, ERBB3, SRC, TGFBR1, MET, ATM, FYN, JAK3
Protein amino acid autophosphorylation	EGFR, TGFBR1, MET, MEKK, FYN, PDGFRA
Enzyme linked receptor protein signaling pathway	FGFR2, ERBB4, ERBB3, SRC, EPHB1, MEKK, AKT2, EGFR, TGFBR1, MET PDGFRA, JAK3
Transmembrane receptor protein tyrosine kinase signaling pathway	EGFR, FGFR2, ERBB4, ERBB3, MET, SRC, EPHB1, PDGFRA, AKT2
Peptidyl-tyrosine phosphorylation	ERBB4, LYN, ERBB3, FYN, PDGFRA, JAK3, SRC, BTK
MAPKKK cascade	EGFR, MEK, TGFBR1, MET, MAPK, MEKK
Peptidyl-tyrosine modification	ERBB4, LYN, ERBB3, FYN, PDGFRA, JAK3, SRC, BTK
Regulation of phosphorylation	EGFR, MEK, LYN, TGFBR1, MET, CDK7, MEKK
Regulation of transferase activity	EGFR, MEK, TGFBR1, MET, CDK7, MEKK
Regulation of phosphate metabolic process	EGFR, MEK, LYN, TGFBR1, MET, CDK, MEKK
Regulation of phosphorus metabolic process	EGFR, MEK, LYN, TGFBR1, MET, CDK, MEKK
Regulation of protein kinase activity	EGFR, MAP2K1, TGFBR1, MET, CDK7, MAP3K5, MAP3K1, MAP2K6
Regulation of kinase activity	EGFR, MAP2K1, TGFBR1, MET, CDK7, MAP3K5, MAP3K1, MAP2K6
Regulation of cell motion	EGFR, LYN, MAP2K1, ERBB4, TGFBR1, MAPK1, MAP3K1, PDGFRA,
Positive regulation of cell motion	EGFR, MAPK1, ERBB4, MAP2K1, LYN, TGFBR1, PDGFRA,
Regulation of cell migration	EGFR, MAPK1, ERBB4, MAP2K1, MAP3K1, PDGFRA,
Cell surface receptor linked signal transduction	FGFR2, ERBB4, ERBB3, SRC, EPHB1, MAP3K1, TEK, EGFR, LYN, TGFBR1, MET, MAPK1, FYN, MAPK14, LCK, PDGFRA, JAK3
Regulation of locomotion	EGFR, MAPK1, ERBB4, MAP2K1, MAP3K1, PDGFRA
Response to hormone stimulus	ERBB4, MAP2K1, LYN, ERBB3, TGFBR1, SRC, MAPK1, PDGFRA
Regulation of MAP kinase activity	EGFR, MAP3K5, MAP2K1, MAP3K1, MET, MAP2K6
Positive regulation of cell communication	EGFR, LYN, ERBB4, ERBB3, TGFBR1, SRC, MAP3K3, LCK
Regulation of cell proliferation	EGFR, FGFR2, ERBB4, LYN, ERBB3, TGFBR1, CDK6, MAPK1, PDGFRA, CSK, MAP2K5
Response to organic substance	EGFR, ERBB4, MAP2K1, LYN, ERBB3, TGFBR1, SRC, MAPK1, FYN, MAPK14, PDGFRA
Response to endogenous stimulus	ERBB4, MAP2K1, LYN, ERBB3, TGFBR1, SRC, MAPK1, PDGFRA
Positive regulation of cell proliferation	EGFR, FGFR2, ERBB4, LYN, TGFBR1, CDK6, MAPK1, PDGFRA, MAP2K5
Positive regulation of cell migration	EGFR, MAPK1, ERBB4, MAP2K1, PDGFRA,
JNK cascade	MAP3K5, MAP3K1, MAP2K4, MAP3K12
Stress-activated protein kinase signaling pathway	MAP3K5, MAP3K1, MAP2K4, MAP3K12
Positive regulation of protein kinase activity	EGFR, MAP3K5, MAP2K1, MAP3K1, TGFBR1, MET, MAP2K6
Positive regulation of locomotion	EGFR, MAPK1, ERBB4, MAP2K1, PDGFRA
Positive regulation of kinase activity	EGFR, MAP3K5, MAP2K1, MAP3K1, TGFBR1, MET, MAP2K6
Positive regulation of MAP kinase activity	EGFR, MAP3K5, MAP2K1, MAP3K1, MET, MAP2K6
Positive regulation of transferase activity	EGFR, MAP3K5, MAP2K1, MAP3K1, TGFBR1, MET, MAP2K6

**Table 2:** Potential novel kinase inhibitors for the treatment of PTC.

Kinase	Kinase inhibitor	Publication number
EGFR	Gefitinib (ZD1839)	3040
	Erlotinib HCl (OSI-744)	4310
	Lapatinib	1681
	Afatinib (BIBW2992)	409
	AZD8931 (Sapitinib)	389
	AG-1478 (Tyrphostin AG-1478)	273
	AG-490 (Tyrphostin B42)	221
	PD153035 HCl	136
	Neratinib (HKI-272)	113
	Canertinib (CI-1033)	94
	Icotinib	69
	AEE788 (NVP-AEE788)	61
	Pelitinib (EKB-569)	43
	Varlitinib	32
	OSI-420	31
	PD168393	24
	Rociletinib (CO-1686, AVL-301)	23
	WZ4002	18
	WHI-P154	15
	CUDC-101	13
	TAK-285	12
	Tyrphostin 9	8
	AST-1306	5
	CL-387785 (EKI-785)	5
	AG-18	2

SRC	Dasatinib	1771
	PP2	345
	Bosutinib (SKI-606)	207
	Saracatinib (AZD0530)	126
	SU6656	64
	KX2-391	8
	NVP-BHG712	2
	Dasatinib Monohydrate	2

MAPK	BMS-536924	29

MET	SU11274	98
	Foretinib (GSK1363089)	26
	SGX-523	7
	NPS-1034	2
	NVP-BVU972	2

ATM	AZD8055	66
	KU-55933 (ATM Kinase Inhibitor)	42
	KU-60019	9

CDK6	Flavopiridol (Alvocidib) HCl	374
	PD-0332991	137
	LY2835219	9
	LDC000067	1

TGFbR1	SB431542	114
	SD-208	25
	SB505124	10
	EW-7197	8
	SB525334	6
	D 4476	2
	RepSox	1
	GW788388	1

PDGFRa	Imatinib (STI571)	6749
	Ponatinib (AP24534)	192
	Nintedanib (BIBF 1120)	102
	Masitinib (AB1010)	52
	Lenvatinib (E7080)	28
	Quizartinib (AC220)	24
	Crenolanib (CP-868596)	19
	Amuvatinib (MP-470)	13
	ENMD-2076	12
	Telatinib	11
	OSI-930	8
	CP-673451	5

ErbB4	AZD8931 (Sapitinib)	12
